# Multimodal Non-Extensive Frequency-Magnitude Distributions and Their Relationship to Multi-Source Seismicity

**DOI:** 10.3390/e26121040

**Published:** 2024-11-30

**Authors:** Erick de la Barra, Pedro Vega-Jorquera, Sérgio Luiz E. F. da Silva

**Affiliations:** 1 School of Business, Universidad Católica del Norte, Coquimbo 1781421, CO, Chile; erick.delabarra@ucn.cl; 2 Department of Physics, Universidad de La Serena, La Serena 1720169, CO, Chile; pvega@userena.cl; 3 Laboratory of Parallel Architectures for Signal Processing, Universidade Federal do Rio Grande do Norte, Natal 59078-900, RN, Brazil; 4 Dipartimento di Scienza Applicata e Tecnologia, Politecnico di Torino, 10129 Torino, Italy; 5 Istituto dei Sistemi Complessi—Consiglio Nazionale delle Ricerche (ISC-CNR), c/o Dipartimento di Scienza Applicata e Tecnologia del Politecnico di Torino, 10129 Torino, Italy

**Keywords:** multimodal seismicity, non-extensive statistical mechanics, q-gamma distributions

## Abstract

We investigate multimodal seismicity by analyzing it as the result of multiple seismic sources. We examine three case studies: the Redoubt and Spurr regions in Alaska, where volcanic and subduction-related seismicity occur, and the Kii Peninsula in Japan, where shallow and deep earthquakes are clearly separated. To understand this phenomenon, we perform spatial, temporal, and magnitude analyses. Our application of non-extensive statistical mechanics shows that multimodal models provide a significantly better fit than unimodal ones. We identify patterns in the distributions of time between events and distances between events using multimodal Tsallis *q*-gamma distributions. In addition, we use the multimodal Sotolongo–Costa model to analyze the magnitude distribution, which effectively captures the complex interactions that may explain the observed lack of fractality in multimodal seismicity.

## 1. Introduction

Earthquakes are complex spatio-temporal phenomena that represent a catastrophic natural hazard, and their triggering mechanisms remain an open question. Indeed, it is not fully understood why earthquakes occur both in tectonically stable parts of the inner regions of tectonic plates (known as intraplate earthquakes [1,2]) and in unstable areas, such as tectonic plate boundaries [3,4] and volcanoes [5,6]. In this context, it is natural to consider various focal mechanisms in the same seismogenic area. Therefore, analyzing the distribution of earthquakes and their magnitudes is crucial for a better understanding of their occurrence and spatial and temporal distribution.

In recent decades, the Gutenberg–Richter (GR) law [7] has been used to describe the distribution of earthquakes by analyzing the number of earthquakes, $N_{>M}$, with a magnitude greater than *M*, as a frequency–magnitude power law. Laboratory studies provide valuable insights into the frequency–magnitude relationship, as described by the GR law, and its variability with different materials and stress conditions. For example, ref. [8] found that microfractures in rocks induced by pressure experiments follow a frequency–magnitude relationship similar to that of earthquakes, with the b-value depending primarily on the stress and the confining pressure. Extending these findings, ref. [9] conducted thermal experiments on rock samples and discovered microquakes generated by thermoelastic stress relief. They observed that the frequency–magnitude relationship of these thermal events had a higher b-value than is typical for ordinary earthquake series. Ref. [10] extended the cumulative frequency–magnitude relationship by reformulating it based on the seismic moment and introducing the b-value as an analog to the original b-value. Finally, ref. [11] investigated the rock behavior under different stress conditions, showing that the confining pressure and intermediate stress strongly influence rock strength and ductility, affecting the frequency and magnitude of seismic events.

However, the GR law exhibits local scale invariance, i.e., the minimum magnitude is not always related to the incompleteness of seismic catalogs [12]. In this respect, the self-similarity of seismic sources and their magnitudes breaks down for low-magnitude earthquakes [13]. Moreover, the statistical distribution of earthquake magnitudes in different areas exhibits multimodal behavior [14,15,16,17] due to the diversity of focal mechanisms in a seismic region. Indeed, multimodality has been associated with non-fractality and the breakdown of self-similarity. For example, multimodal seismicity patterns have been observed in mining-induced seismicity, attributed to complex geologic features associated with inhomogeneous fault planes and dikes [18]. In addition to anthropogenic cases, natural features also exhibit multimodal characteristics, as in the case of the well-known Himalayan seismicity due to the non-planar and frictional geometry of the main Himalayan thrust, which is responsible for triggering numerous earthquakes [19,20].

In addition, several studies have demonstrated the existence of long-range correlations between seismic events, i.e., earthquake sequences exhibit a significant statistical dependence between two distant events, as measured by the autocorrelation function [21,22,23]. Significant progress has been made in seismological modeling through the use of Tsallis *q*-statistics (also known as non-extensive statistical mechanics) by incorporating concepts of long-range correlation [24,25,26]. More specifically, the Sotolongo–Costa–Posadas (SCP) model is based on a probability distribution derived from the principle of maximum entropy applied to the Tsallis entropy. This model generalizes the GR law and physically justifies the relationship between frequency and earthquake magnitude [27,28,29]. Based on the theory of seismic moment scaling (see, e.g., [30]), Silva et al. [31] slightly revised the SCP model by introducing a more realistic link between seismic energy and fragment size. The SCP model provides a physically based approach that takes into account not only the rough surfaces of the fault planes but also the distribution of rock fragments between plates and the theory of seismic moment scaling. In this sense, the SCP model assumes that the physical mechanisms for triggering an earthquake are related to the distribution of fragments and the energy distribution of the earthquake, which is determined by maximizing the Tsallis *q*-entropy according to first principles. Subsequently, a number of studies have shown the universality of the SCP model in analyzing seismicity in various regions [32,33,34,35,36]. However, all these studies and related works focused on the unimodal analysis of the statistical distribution of earthquake magnitudes.

In this work, we consider a multimodal extension of the SCP model presented in [37] and multimodal versions of Tsallis *q*-gamma distributions to analyze three seismogenic regions that exhibit multimodal empirical distributions. In this effort, we investigate the transferability of multimodal non-extensive models to describe multimodal and non-fractal seismicity by explaining the multi-source seismic mechanisms and their characteristics in temporal and spatial terms. This paper is organized as follows. In Section 2, we present the models used in seismology and their non-extensive multimodal extensions based on the SCP and Tsallis q-distributions. In Section 3, we describe the main features of the real-world dataset used. Then, we devote Section 4 to the presentation of the results to demonstrate the robustness of our proposed model in analyzing the seismicity generated by multi-source configurations. Finally, in Section 5, we summarize the concluding remarks of this work.

## 2. Multi-Source Seismicity and Bimodal Distributions in the Non-Extensive Approach

In this section, we present a multimodal extension of the SCP model to describe multi-source seismicity. The bimodal extension of the SCP model has recently been successfully used to describe the anthropogenic seismicity triggered by the exploitation of underground mines [37]. Two types of earthquakes can be distinguished in anthropogenic seismicity: one is triggered directly by human activity and is of low energy, while the other is triggered by tectonic stresses released by human activity and is stronger than quakes triggered directly by mining operations. Each type of earthquake is associated with different modes in the multimodal SCP model. In the cases to be analyzed, two groups of earthquakes are investigated, namely shallow and deep events, which are easily identified by their spatial distribution.

Our aim is to study the cumulative frequency–magnitude distribution (FMD) of earthquakes, the spacing between events, and the recurrence times. To analyze the last two objectives, we introduce bimodal extensions to the Tsallis q-distributions, which are commonly used to describe the distances and times between events.

### 2.1. Bimodal Non-Extensive Model for Earthquakes

Based on non-extensive statistical mechanics (or Tsallis q-statistics), Sotolongo, Costa, and Posadas [27] introduced a new fragment-asperity interaction model for modeling earthquake magnitude distributions. This model consists of two fault planes, with rough surfaces and irregular rock fragments occupying the space between them. In this regard, the statistical distribution of fragments maximizes the Tsallis *q*-entropy

(1) $$\max_{p}\;S_{q}\left[p\right]\;/k_{B}=\frac{1}{q-1}\left(1-\int_{0}^{\infty }p^{q}\left(\sigma \right)d\sigma \right),$$

subject to

(2) $$\int_{0}^{\infty }p\left(\sigma \right)d\sigma =1,$$

(3) $$\langle \sigma_{q}\rangle =\int_{0}^{\infty }\sigma P_{q}\left(\sigma \right)d\sigma ,\;\mathrm{with},\;\;P_{q}\left(\sigma \right)=\frac{p^{q}\left(\sigma \right)}{\int_{0}^{\infty }p^{q}\left(\sigma \right)d\sigma },$$

conditions, where $k_{B}$ is the Boltzmann constant, p(σ) denotes the probability density function related to the fragment size σ, and q∈R is the non-extensivity parameter (entropic index). The q-parameter allows the escort distribution to scan the structure of p(σ) differently, emphasizing events with higher or lower probabilities depending on *q*. The constraints in Equation (2) and Equation (3) are, respectively, the normalization condition and the *q*-expectation value [38], in which $P_{q}\left(\cdot \right)$ represents the escort probability function [39,40]. Escort distributions are derived probability functions that allow a controlled modification of the original distribution p(σ) to emphasize different aspects of the probability structure. In the context of Tsallis q-statistics, these escort probability functions arise naturally when maximizing the Tsallis q-entropy. A notable example is the q-generalized Gaussian distribution, which emerges as an equilibrium distribution from this maximization [41,42,43]. In the field of source coding, for instance, escort distributions are related to Campbell’s coding theorem by associating generalized length measures with the Rényi–Tsallis entropy [44]. We invite the reader to refer to [45,46,47,48,49] for an exploration of the fundamental concepts and diverse applications of the escort probability function.

The optimization of Equation (1), subject to the constraints in (2), leads to the distribution for the following fault plane-related fragments [31]:

(4) $$p\left(\sigma \right)={\left[1-\left(\frac{1-q}{2-q}\right)\left(\sigma -\langle \sigma_{q}\rangle \right)\right]}^{\frac{1}{1-q}},$$

valid for 1<q<2. By considering an earthquake energy ε to be proportional to $r^{3}$, where *r* is the linear dimension of fragments filling the gap between the sliding plates, we have that $\sigma -\langle \sigma_{q}\rangle ={\left(\varepsilon /\alpha \right)}^{2/3}$ [31], where α is a proportionality constant. Moreover, it is noteworthy that the relation between the magnitude *m* of a seismic event and its released seismic energy is given by [30] $m=\frac{2}{3}log_{10}\left(\varepsilon \right)$. Thus, we may write, after some algebra, the non-extensive GR law as [31,50]:

(5) $$\log_{10}\left(N_{>M}\right)=\log_{10}\left(N\right)+\left(\frac{2-q_{M}}{1-q_{M}}\right)\log_{10}\left[\frac{1-\left(\frac{1-q_{M}}{2-q_{M}}\right)\frac{10^{M}}{\alpha^{2/3}}}{1-\left(\frac{1-q_{M}}{2-q_{M}}\right)\frac{10^{M_{0}}}{\alpha^{2/3}}}\right],$$

for $M\geq M_{0}$, or equivalently,

(6) $$\log_{10}\left(N_{>M}\right)=a_{q}-b_{q}\left(\log_{10}\left[1+\left.10^{M}/\left(b_{q}\cdot \alpha^{2/3}\right)\right.\right]-\log_{10}\left[1+\left.10^{M_{0}}/\left(b_{q}\cdot \alpha^{2/3}\right)\right.\right]\right),$$

where $a_{q}=\log_{10}\left(N\right)$, $b_{q}=\frac{2-q_{M}}{q_{M}-1}$ is the generalized *b*-value, and $q_{M}$ is the entropic index related to the frequency–magnitude distribution.

The multimodal behavior of the F–M distribution (FMD) is observed in natural earthquakes. It can be explained by multi-source seismicity, as in cases where many plates, the conjunction of plates, or volcanoes are present. Thus, each source generates different types of tremors, which break the self-similarity in the FMD. As in anthropogenic tremors in mining operations, the multimodal GR distribution can be used to describe multi-source natural earthquakes, that is, a piecewise linear function for the survival $\log_{10}N_{>M}$ can be proposed:

$$\log_{10}\left(N_{>M}\right)\approx \left\{\begin{array}{cc} a_{1}-b_{1}\left(M-M_{0}\right), & M_{0}\leq M\leq M_{T},\\ a_{2}-b_{2}\left(M-M_{T}\right), & M\geq M_{T}, \end{array}\right.$$

But, the multimodal GR ignores the long-range effects, and a multimodal extension of (5) would be suitable to describe multi-source seismicity considering autocorrelation and fractality:

(7) $$\log_{10}\left(N_{>M}\right)=\left\{\begin{array}{cc} a_{1}-b_{1}\left(\log_{10}\left[1+\frac{10^{M}}{b_{1}\cdot \alpha_{1}^{2/3}}\right]-\log_{10}\left[1+\frac{10^{M_{0}}}{b_{1}\cdot \alpha_{1}^{2/3}}\right]\right), & M_{0}\leq M\leq M_{T}\\ a_{2}-b_{2}\left(\log_{10}\left[1+\frac{10^{M}}{b_{2}\cdot \alpha_{2}^{2/3}}\right]-\log_{10}\left[1+\frac{10^{M_{T}}}{b_{2}\cdot \alpha_{2}^{2/3}}\right]\right), & M\geq M_{T}, \end{array}\right.$$

where the continuity conditions must be satisfied:

(8a) $$a_{1}=\log_{10}N_{>M_{0}},\;\mathrm{and}$$

(8b) $$a_{2}=a_{1}-b_{1}\left(\log_{10}\left[1+\left.10^{M_{T}}/\left(b_{1}\cdot \alpha_{1}^{2/3}\right)\right.\right]-\log_{10}\left[1+\left.10^{M_{0}}/\left(b_{1}\cdot \alpha_{1}^{2/3}\right)\right.\right]\right).$$

The bimodal Sotolongo–Costa density is also derived:

$$f\left(x\right)=\left\{\begin{array}{cc} \frac{b_{1}\ln10}{b_{1}\alpha_{1}^{2/3}+10_{0}^{M}}{\left(\frac{\left[1+\frac{10^{M}}{b_{1}\cdot \alpha_{1}^{2/3}}\right]}{\left[1+\frac{10^{M_{0}}}{b_{1}\cdot \alpha_{1}^{2/3}}\right]}\right)}^{-b_{1}-1}10^{M}, & M_{0}\leq M\leq M_{T}\\ \frac{b_{2}\ln10}{b_{2}\alpha_{2}^{2/3}+10_{0}^{M}}{\left(\frac{\left[1+\frac{10_{T}^{M}}{b_{1}\cdot \alpha_{1}^{2/3}}\right]}{\left[1+\frac{10^{M_{0}}}{b_{1}\cdot \alpha_{1}^{2/3}}\right]}\right)}^{-b_{1}-1}{\left(\frac{\left[1+\frac{10^{M}}{b_{2}\cdot \alpha_{2}^{2/3}}\right]}{\left[1+\frac{10^{M_{0}}}{b_{2}\cdot \alpha_{2}^{2/3}}\right]}\right)}^{-b_{2}-1}10^{M}, & M\geq M_{T}, \end{array}\right.$$

A trimodal distribution can be defined analogously.

The application of the bimodal extension to earthquake distributions assumes the existence of two modes, with each mode following a Sotolongo–Costa distribution. At the same time, the Sotolongo–Costa model describes non-linearity, long-range interactions, fractality, self-organized criticality, long memory effects, and scaling in seismicity because it is based on non-extensive statistical mechanics, providing a consistent statistical framework for the description of seismicity and its properties [33,34].

### 2.2. Bimodal Tsallis Distribution and Interevent Distances

The Tsallis distribution is used to model three-dimensional distances between the hypocenters of successive earthquakes. The unimodal Tsallis distribution is defined as [51]

(9) $$p\left(x\right)=\left(2-q_{TS}\right)b{\left(1+b_{TS}\left(q_{TS}-1\right)\left(x-x_{0}\right)\right)}^{1/(1-q_{TS})}.$$

The subscript TS denotes that the parameters are from the Tsallis distribution but it will be omitted when the context is clear. Here, b>0, $x\in R^{+}$ if 1<q<2, $x\in (0,x_{\max})$ if q<1, and x∈C with $\int_{C}p<\infty$ if q>2. Additionally, the Tsallis model appears in the interevent times modeling, that is, the elapsed time between successive tremors.

Given that, in some cases, the magnitudes behave bimodally or trimodally, we expect to observe similar behavior for the interevent distances and interevent times in these cases.

In order to model the multimodality of the interevent distances and interevent times, we define the bimodal Tsallis distribution by

$$f\left(x\right)=\left\{\begin{array}{cc} \left(2-q_{1}\right){\left(1+\left(q_{1}-1\right)b_{1}\left(x-x_{0}\right)\right)}^{\frac{1}{1-q_{1}}}, & x_{0}\leq x\leq x_{T}\\ k\left(2-q_{2}\right){\left(1+\left(q_{2}-1\right)b_{2}\left(x-x_{T}\right)\right)}^{\frac{1}{1-q_{2}}}, & x\geq x_{T}, \end{array}\right.$$

and integrating, we obtain

$$\bar{F}\left(x\right)=\left\{\begin{array}{cc} {\left(1+\left(q_{1}-1\right)b_{1}\left(x-x_{0}\right)\right)}^{\frac{2-q_{1}}{1-q_{1}}}, & x_{0}\leq x\leq x_{T}\\ k{\left(1+\left(q_{2}-1\right)b_{2}\left(x-x_{T}\right)\right)}^{\frac{2-q_{2}}{1-q_{2}}}, & x\geq x_{T}, \end{array}\right.$$

where $\bar{F}\left(x\right)=P\left(X>x\right)$ is the survival function and *k* is determined by the continuity condition at $x=x_{T}$:

$$k={\left(1+\left(q_{1}-1\right)b_{1}\left(x_{T}-x_{0}\right)\right)}^{\frac{2-q_{1}}{1-q_{1}}}.$$

As usual, it is preferable to plot $\log_{10}\left(N_{>x}\right)$ instead P(X>x), where $N_{>x}$ is the number of data greater than *x*, because the logarithm smooths out the differences along the curve P(X>x):

(10) $$\log_{10}\left(N_{>x}\right)=\left\{\begin{array}{cc} A_{1}-B_{1}\log_{10}\left(1+\left(q_{1}-1\right)b_{1}\left(x-x_{0}\right)\right), & x_{0}\leq x\leq x_{T}\\ A_{2}-B_{2}\log_{10}\left(1+\left(q_{2}-1\right)b_{1}\left(x-x_{T}\right)\right), & x\geq x_{T}, \end{array}\right.$$

where $A_{1}=\log_{10}N$, $B_{i}=\left(2-q_{i}\right)/\left(q_{i}-1\right)$ for i=1,2, *N* is the number of data, and $A_{2}=\log_{10}Nk=A_{1}-B_{1}\log_{10}\left(1+\left(q_{1}-1\right)b_{1}\left(x_{T}-x_{0}\right)\right)$.

### 2.3. q-Gamma Distributions and Interevent Times

Understanding the time occurrence between successive earthquakes is vital for estimating seismic hazard. Several models have been developed to describe the interevent time distribution of earthquakes, which can be classified into Poissonian and non-Poissonian models. Non-Poissonian models include the Tsallis distribution [33] and the gamma distribution [34,52,53]. Here, we propose to characterize multimodal seismicity using the unimodal-bimodal versions of the Tsallis distribution (10) and the *q*-gamma distribution, a deformed version of the gamma distribution, which is given by

$$f\left(t\right)=c{\left(t-t_{0}\right)}^{\gamma_{\Gamma }-1}{\left(1+\left(q_{\Gamma }-1\right)b_{\Gamma }\left(t-t_{0}\right)\right)}^{\frac{1}{1-q_{\Gamma }}},\;t>t_{0}$$

The subscript Γ denotes that the parameters are from the *q*-gamma distribution, but it will be omitted when the context is clear. Here, $t\in R^{+}$, 1<q<2 is the parameter of deformation, b,γ≥0 are shape parameters, and *c* is a normalization constant given by [54]

(11) $$c=\frac{{\left[(q-1)b\right]}^{\gamma }}{B\left(\gamma ,\frac{1}{q-1}-\gamma \right)}.$$

Since the interevent distribution follows a gamma distribution, it is reasonable to propose the non-extensive version of this distribution to model the interevent times, that is, the *q*-gamma distribution. It is worth noting that the *q*-gamma law is a generalization of the Tsallis distribution, which has been used to describe interevent time data [33]. The cumulative distribution function for 1<q<2 can be expressed in terms of the incomplete beta function *I* [54]:

$$F\left(x;q,b,\gamma ,x_{0}\right)=I\left(\frac{\left(q-1\right)b\left(x-x_{0}\right)}{\left(q-1\right)b\left(x-x_{0}\right)+1};\gamma ,\frac{1}{q-1}-\gamma \right)$$

Note that for q→1, the *q*-gamma distribution becomes the gamma distribution. Given that multi-source seismicity is being studied, it is reasonable to expect bimodal behavior for the interevent time distribution. Its modeling is especially important in order to properly estimate the hazard function, that is, the probability that at least one earthquake with a magnitude greater than *M* will occur in the next time interval ΔT, if the previous one occurred before time *T* [33].

For the interevent time distribution in multi-source seismicity, we propose the bimodal *q*-gamma distribution:

(12) $$f\left(t\right)=\left\{\begin{array}{cc} c_{1}{\left(t-t_{0}\right)}^{\gamma_{1}-1}{\left(1+\left(q_{1}-1\right)b_{1}\left(t-t_{0}\right)\right)}^{\frac{1}{1-q_{1}}}, & t_{0}\leq t\leq t_{T}\\ c_{2}{\left(t-t_{T}\right)}^{\gamma_{2}-1}{\left(1+\left(q_{2}-1\right)b_{2}\left(t-t_{T}\right)\right)}^{\frac{1}{1-q_{2}}}, & t\geq t_{T}, \end{array}\right.$$

where $1<q_{1},q_{2}<2$, $b_{1},b_{2},\gamma_{1},\gamma_{2}\geq 0$. $c_{1}$ takes the form (11), and $c_{2}$ is such that the continuity condition is satisfied at $x=x_{T}$. An important form of (12) is the logarithm of the number of events whose interevent times are greater than time *t*:

$$\log_{10}\left(N_{>t}\right)=\left\{\begin{array}{cc} A_{1}-\log_{10}\left(1-F(t;q_{1},b_{1},\gamma_{1},t_{0})\right), & t_{0}\leq t\leq t_{T}\\ A_{2}-\log_{10}\left(1-F(t;q_{2},b_{2},\gamma_{2},t_{T}))\right), & t\geq t_{T}, \end{array}\right.$$

where $A_{1}=\log_{10}N$ and $A_{2}=\log_{10}N+\log_{10}\left(1-F(t_{T};q_{1},b_{1},\gamma_{1},t_{0})\right)$.

According to this, the interevent times can be modeled with a very good fit using the bimodal *q*-gamma distribution, as we will see in Section 4 (see Table 5).

## 3. Data and Case Studies

**Figure 1:**
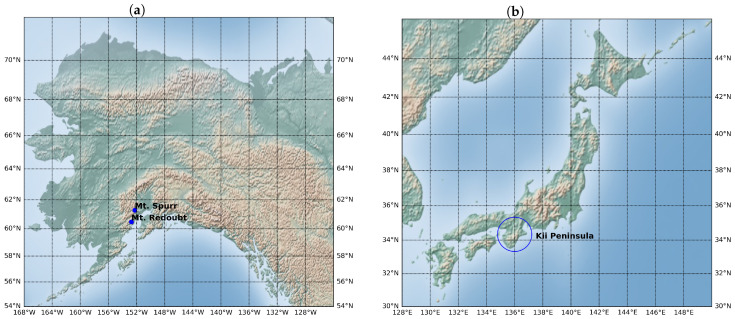
Geographical locations of studied seismogenic regions. In (**a**), the coordinates of the Alaskan volcanoes, Mt. Redoubt and Mt. Spurr, are marked by blue dots, and in (**b**), the Japanese Kii Peninsula is represented by a blue circle.

**Figure 2:**
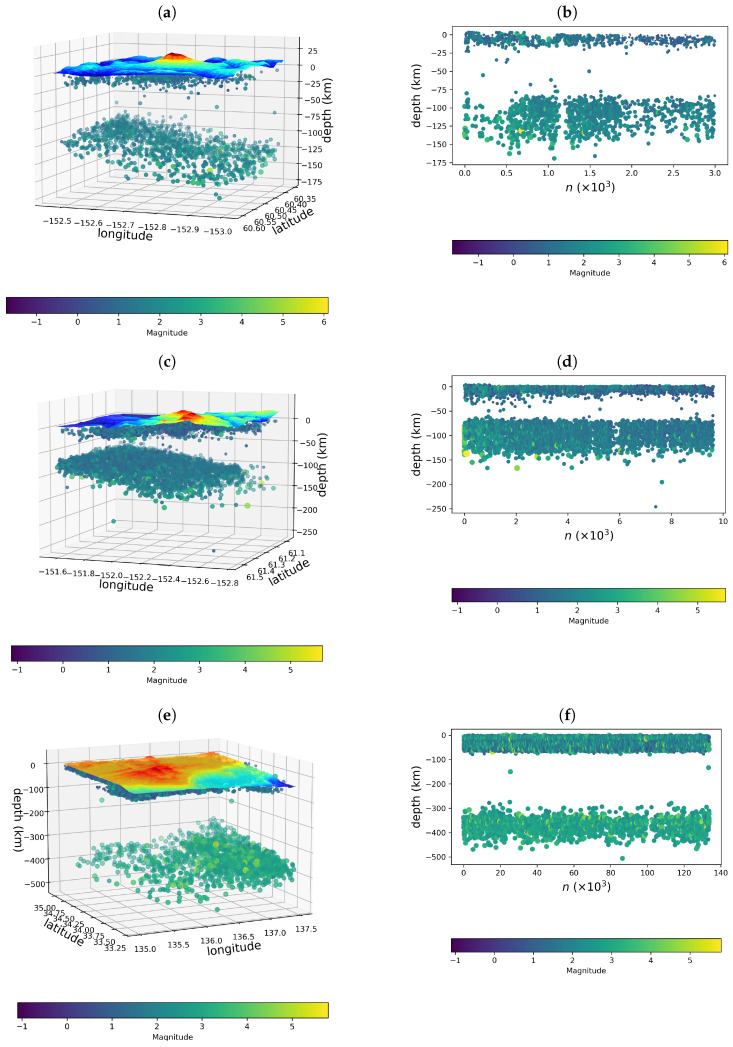
Three-dimensional base maps representing the distribution of earthquakes in the (**a**) Mount Redoubt, (**c**) Mount Spurr, and (**e**) Kii Peninsula regions. The green points represent the hypocenters, while the colored surfaces represent the topographic maps. Topographic data were multiplied by a factor of ten for better visualization. Panels (**b**,**d**,**f**) show the temporal development in counts of the hypocenter depth distributions associated with the Mount Redoubt (1973–2022), Mount Spurr (1970–2022), and Kii Peninsula (2009–2020) regions.

To experimentally evaluate our proposal, we consider three seismogenic areas (Mount Redoubt, Mount Spurr, and the Kii Peninsula), using earthquakes from two catalogs: the United States Geological Survey (USGS) and the Japan Meteorological Agency (JMA), as shown in Table 1. Mount Redoubt (60.4852 N, 152.7431 W) and Mount Spurr (61.2997 N, 152.2514 W) are active stratovolcanoes in the Alaskan volcanic zone, while the Kii Peninsula is a large Japanese peninsula (34.1810 N, 135.5718 E). Data for the Spurr and Redoubt volcanoes were obtained from the USGS website, while data for the Kii Peninsula were obtained from the JMA. Table 1 summarizes the time periods and number of events analyzed in this study, and Figure 1 shows the locations of these regions on a map. These regions were selected because they exhibit spatially differentiated seismicity that is consistent over time, as shown in Figure 2. We hypothesize that the clustering of earthquakes in these areas follows different patterns, i.e., the FMDs differ for each cluster in a given area. Nevertheless, we need to analyze the time windows for which the degree of detection is constant, i.e., the degree of completeness must be constant. The selected volcanic areas are significantly smaller than the Kii area, as seismicity is particularly noticeable in the vicinity of the volcanoes due to volcanic activity. The selected time periods were chosen to ensure constant completeness values during the periods investigated. This criterion is intended to avoid mixing data with different degrees of completeness, which could lead to bimodal behavior. The irregularity of $M_{c}$ in the volcanic areas compared to the behavior of $M_{c}$ on the Kii Peninsula explains the difference in size of the selected time windows in which seismicity is analyzed. Both Mt. Redoubt and Mt. Spurr are active stratovolcanoes, but Mt. Redoubt has shorter intervals between eruptions, producing more frequent seismological records. This difference explains why the years analyzed vary between the Redoubt and Spurr areas.

Figure 2a,b show 3D base maps depicting the distribution of earthquakes in Mount Redoubt, Mount Spurr, and the Kii Peninsula, in which the green points represent the hypocenters, while the colored surfaces represent the topographic maps. In these figures, we can see that deep earthquakes are spatially separated from shallow earthquakes in all seismic regions. The deep earthquakes occur at depths ranging from 80 to 150 km, 60 to 150 km, and 300 to 450 km, in the Mount Redoubt, Mount Spurr, and Kii regions, respectively, while shallow events occur at depths less than 20, 25, and 70 km. Deep hypocenters correspond to tectonic earthquakes, often large, while shallow earthquakes are generally low-magnitude events. In the figures, panels (d)–(f) show the temporal distributions of the earthquakes illustrated in panels (a)–(c), where the data points refer to all seismic events, sorted according to the occurrence time index n in ascending order, with n = 1 being the first detected earthquake, n = 2 being the second one, and so on. In fact, the existence of a region free of tremors is remarkable and separates the shallow and deep events.

It is worth noting that these three seismogenic regions are known to experience multi-source seismic activity for different reasons, and therefore, multimodal statistical behavior is expected. For instance, Mounts Redoubt and Spurr are located at the Aleutian Arc, where the shallow events can be explained by volcanic activity close to the surface at the volcanic arc, which is induced by subduction of the Pacific Plate (oceanic crust) beneath the North American Plate (continental crust) [55]. As the oceanic plate is subducted, the pressure and temperature increase, turning the rocks into magma. The magma ascends as a result of partial melting and interaction with the mantle wedge, forming magma chambers whose contents are expelled through violent eruptions. The deep earthquakes in the Mount Redoubt and Mount Spurr regions are triggered by the subduction of the Pacific Plate beneath the North American Plate [55]. In the Kii Peninsula case, the multimodal seismicity feature results from several factors, such as a reflection of the Median Tectonic Line seismic activity, which extends 900 km in an east–west direction, and the oblique subduction of the Philippine Sea plate beneath the Southwest Japan Arc through the Nankai Trough [56]. Thus, we can observe long-continuing swarm activities of shallow, slow, and megathrust earthquakes [57]. Although our analysis considers shallow events whose hypocenters are located in the interval [0,70] km, these events can be subclassified as shallows with a focus in the interval [0,20] km and deep events with a focus in the interval [20,70] km, as discussed in [56]. But our analysis of Kii earthquakes focuses only on two groups of events: those with a hypocenter in the depth interval [0,70] km and those with a hypocenter in [300,450] km.

**Figure 3:**
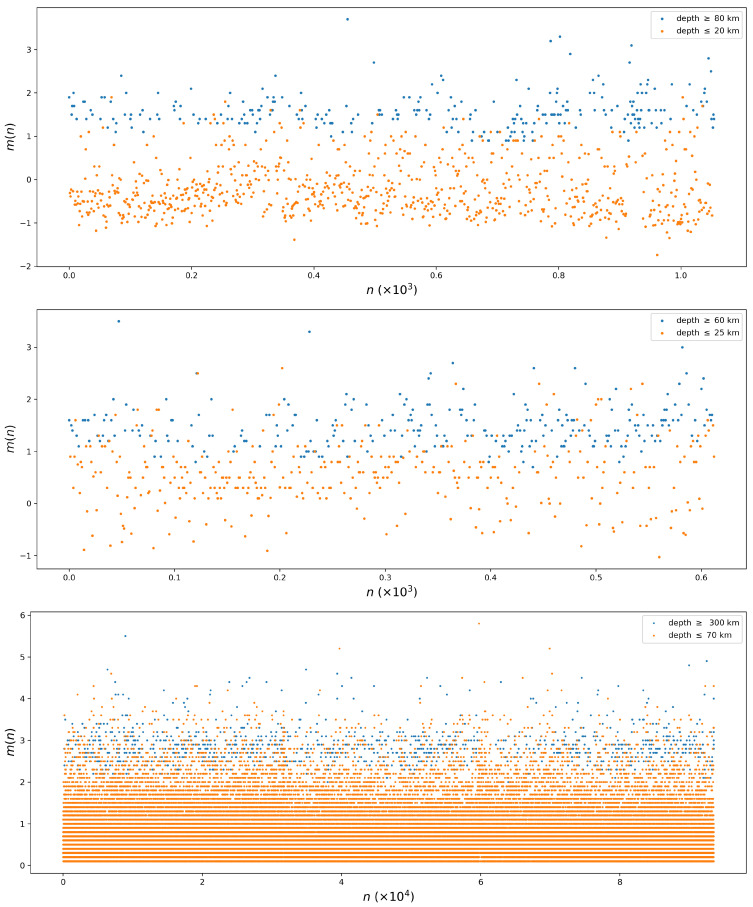
Earthquake time series. On the *x*-axis, *n* represents the number of events, ordered increasingly, and on the *y*-axis, m(n) represents the magnitude of the event *n*. In the first, second, and third rows, we show the time series for the Redoubt, Spurr, and Kii regions, respectively. Shallow events are depicted in orange, and deep events are depicted in blue. Clearly, event magnitudes are generally larger for deep events than for shallow ones.

**Figure 4:**
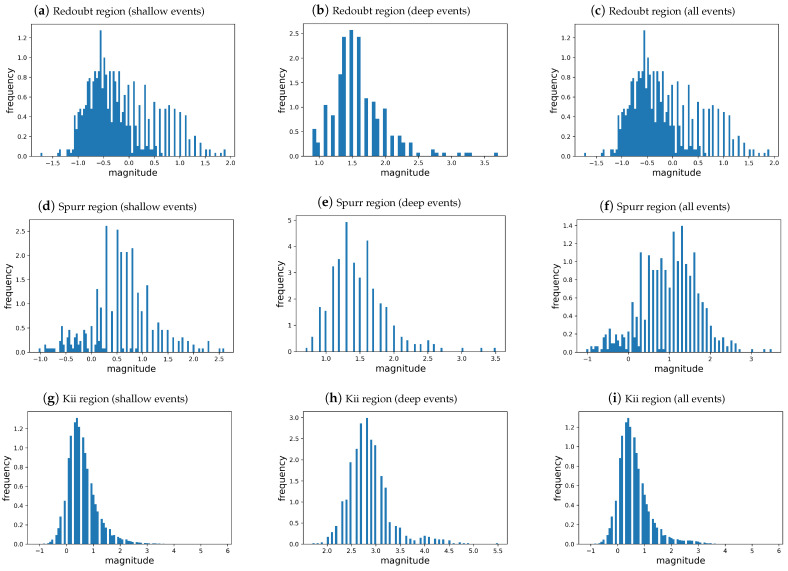
Histogram of the magnitudes of earthquakes. The *x*-axis represents the magnitude and the *y*-axis represents the frequency. In the first, second, and third rows, we show the histograms for the Redoubt, Spurr, and Kii regions, respectively. In the third column, the magnitude distributions for all events are shown. Histograms for shallow events are depicted in the first column, and histograms for deep events are shown in the second column.

To visually examine the impact of shallow and deep earthquakes on the entire dataset, we analyzed the time series of earthquake magnitudes m(n), sorted according to the occurrence time index *n* in ascending order, with m(1) representing the first recorded earthquake, m(2) representing the second, and so on, as depicted in Figure 3 for the Redoubt, Spurr, and Kii areas. In Figure 3, shallow events are represented by orange dots, while deep events are represented by blue dots. We can see that shallow earthquakes are concentrated at low magnitudes, while deep events significantly contribute to the concentration of events around magnitudes of 1.5, 1.5, and 3 for the Redoubt, Spurr, and Kii Peninsula regions, respectively. This contribution from deep events suggests the generation of a second statistical mode, which will be discussed later. Furthermore, at least two modes are observed in the histograms presented in Figure 4. In the magnitude distribution of shallow events (first column of Figure 4), frequencies concentrate at small magnitudes, while for deep events (second column of Figure 4), the probability accumulates at higher magnitudes. The third column of Figure 4 shows the histograms resulting from the superposition of the magnitude distributions for shallow and deep events, leading to bimodal distributions. In the histograms for the Redoubt and Spurr events (Figure 4c,f), shallow events are identified by bold bars, as they are more frequent than deep events. By observing the histograms for all events, we can visually identify at least two modes for the Redoubt region, which are barely visible for the Spurr region and not identifiable for the Kii region. The difficulty in visually observing the modes, especially for the Kii region, is explained by the small proportion of deep events (see Table 1); in this case, Figure 3 clearly shows the existence of at least two modes for that region.

## 4. Results

Before analyzing the portability of our proposed model, we examined the magnitude distributions for all seismic catalogs to explore the data consistency and completeness by determining the completeness magnitude. The completeness magnitude $M_{c}$ is the minimum magnitude above which all earthquakes within a certain volume are reliably detected, avoiding inconsistency issues. Since space-differentiated earthquake clusters are present in the three regions studied in this work, we independently determined the completeness magnitude for each cluster using the method described in [54], which is based on the Maximum Curvature Method [58]. In each studied region, two clusters of earthquakes were observed: shallow events and deep ones. The completeness magnitude was determined for each cluster in a region, and the data were filtered, retaining the events whose magnitudes were above the $M_{c}$. We then obtained accurate measurements by combining the relevant data from each cluster [58]. The obtained completeness magnitudes are shown in Table 2. In this table, we observe that $M_{c}$ for shallow events is less than $M_{c}$ for deep quakes, which was expected because detection is more accurate for events that occur close to seismographs.

The fits of the FMD and the interevent distance and time distributions were performed using the curve_fit module in Python from the scipy.optimize package, employing the Trust Region Reflective algorithm. In addition, the confidence intervals for the estimated parameters were calculated using this function.

**Figure 5:**
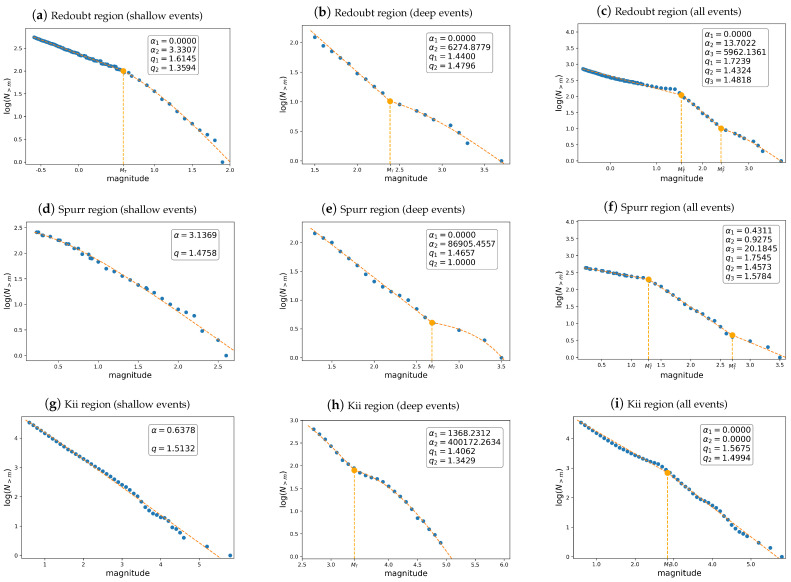
FMDs for the Redoubt, Spurr, and Kii regions. The first, second, and third columns correspond to shallow, deep, and all events. In each graphic, the empirical and multimodal SCP-fitted distributions are shown. The standard errors of the parameters are shown in Table 4. The main measures of goodness of fit are shown in Table 5, revealing very tight fits.

Figure 5 depicts the FMD as a function of the magnitudes for all regions examined. The dashed orange curve represents the best fit based on our proposed model, while the dots represent the data points. Based on a visual examination, we propose a multimodal model. The first, second, and third rows refer to the Mount Redoubt, Mount Spurr, and Kii Peninsula regions, respectively, while the first, second, and third columns correspond to shallow, deep, and all (shallow + deep) events. When examining shallow events, bimodal FMD distributions can be observed around the active volcano Mount Redoubt, but for the Mount Spurr and Kii regions, unimodal behavior of the FMD can be observed. For the Mount Redoubt region, note the high density and small α-value ($\alpha_{1}=0$) for tremors with a magnitude less than $M_{T}=0.6$. and the low density and relatively large α-value ($\alpha_{2}=3.3307$) for events with a magnitude above $M_{T}=0.6$. Thus, in the Mount Redoubt area, numerous low-energy events associated with swarms can be described by the first section of the SCP bimodal model. Similar behavior is observed for deep events in the three analyzed areas: the first section of the SCP bimodal distribution presents a relatively small α-value, which, in the classical SCP model, means low-energy events. In contrast, the second section exhibits a high α-value, which means high-energy events. Additionally, in the case of deep events, the α-values for the Redoubt and Spurr regions are significantly lower than the α-values corresponding to the Kii area, as expected, given that the earthquakes in the Alaskan regions are considerably smaller than those in the Kii Peninsula. We note that multimodal models arise from the presence of multiple sources of seismicity, such as anthropogenic and natural origins, or from sets of earthquakes with different characteristics (e.g., both deep and shallow earthquakes). However, our data also support the existence of multiple modes, even within sets of events that display a single characteristic. For example, the deep events shown in the second column of Figure 5 and the shallow events in the Redoubt area depicted in Figure 5a suggest that interactions between deep and shallow events can generate new modes within a set of events that otherwise share a common characteristic. This is reasonable because deep and shallow events are not independent earthquake sets.

Table 3 shows the errors of the unimodal and multimodal fits for the FMD curve. As can be seen, the fit for the multimodal models is tighter than that for the unimodal ones. Additionally, the standard error of each parameter is shown in this table.

**Figure 6:**
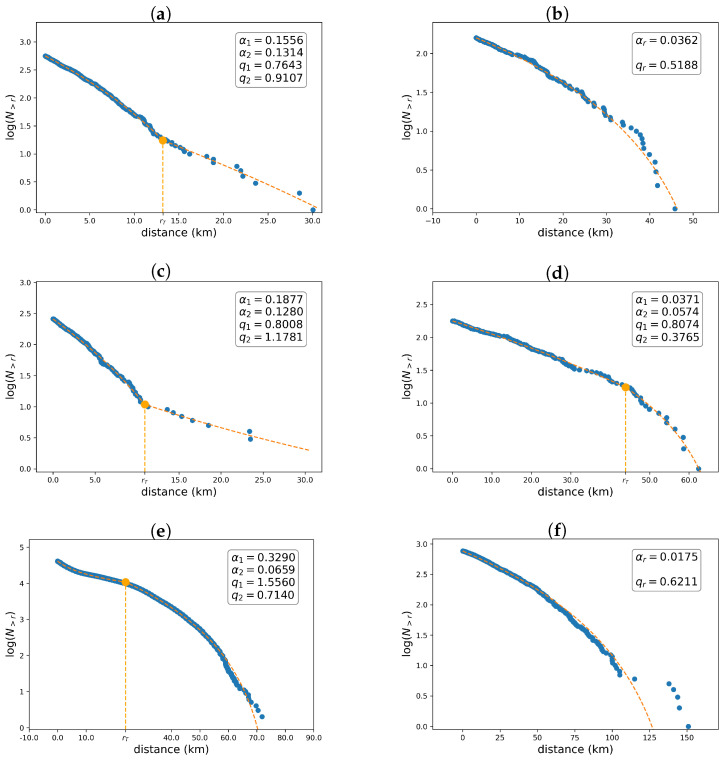
Interevent distance distributions. The first, second, and third rows correspond to the distributions for the Redoubt, Spurr, and Kii regions. In the first and second columns, the distributions for shallow and deep events are shown, respectively. The fitted curves are the unimodal and bimodal Tsallis distributions. The standard errors of the parameter are shown in Table 6. The main measures of goodness of fit are shown in Table 5, revealing very tight fits.

Figure 6 depicts the interevent distance distributions, where the dashed orange curve represents the best fit, while the blue dots represent the data points. The first, second, and third rows correspond to the Mount Redoubt, Mount Spurr, and Kii Peninsula regions, respectively, while the first and second columns correspond to shallow and deep events, respectively. The results reveal that the interevent distances for shallow events present similar behavior for the Mount Redoubt and Spurr volcanic areas, while the curves for the three areas are similar when all events are considered, i.e., shallow and deep ones. When comparing Figure 6a,c, we observe that the interevent distance distribution initially exhibits a high slope, followed by a smaller slope. The α-values are similar, with an order of ∼$10^{-1}$. Most *q*-values are less than one, which is usually observed in unimodal seismicity [33]. Nevertheless, for shallow events in the Kii peninsula, the behavior is different because distances less than $r_{T}=23.9$ km are governed by an entropy index greater than one, while distances greater than $r_{T}$ are described by a *q*-value less than one, as usual. The complex behavior of shallow quakes may result from the interaction between shallow and deep earthquakes. For deep events, the behavior of the interevent distance distribution is similar to the standard results found in the literature [33] for the three analyzed regions: the curves are well described by a unimodal or a bimodal Tsallis distribution, and the *q*-values are less than one.

**Figure 7:**
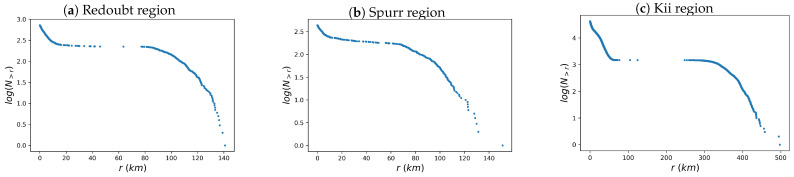
Interevent distance distributions considering all events (both shallow and deep ones) above the completeness magnitude. In (**a**–**c**), the distributions for the Redoubt, Spurr, and Kii regions are depicted.

In Figure 7. we show the interevent distance distribution considering all events above $M_{0}=M_{c}$, i.e., shallow and deep ones. A convex curve first appears in the distribution, followed by a plateau region and then a concave curve. The convex section corresponds to successive events that occur in the near field. The plateau section describes successive earthquakes occurring at different depths; that is, one is shallow and the other is deep, or both are at similar depths (i.e., both are shallow or both are deep). Note that the plateau section appears in the interval distances from 20 to 80 km, 20 to 60 km, and 60 to 300 km for the Redoubt, Spurr, and Kii regions, respectively. These intervals include the distances that separate the shallow and deep quakes, which are 60, 35, and 230 km for the Redoubt, Spurr, and Kii regions, respectively. The concave section represents successive earthquakes occurring at different depths because it starts at a distance greater than the maximum interevent distance for each cluster of earthquakes.

**Figure 8:**
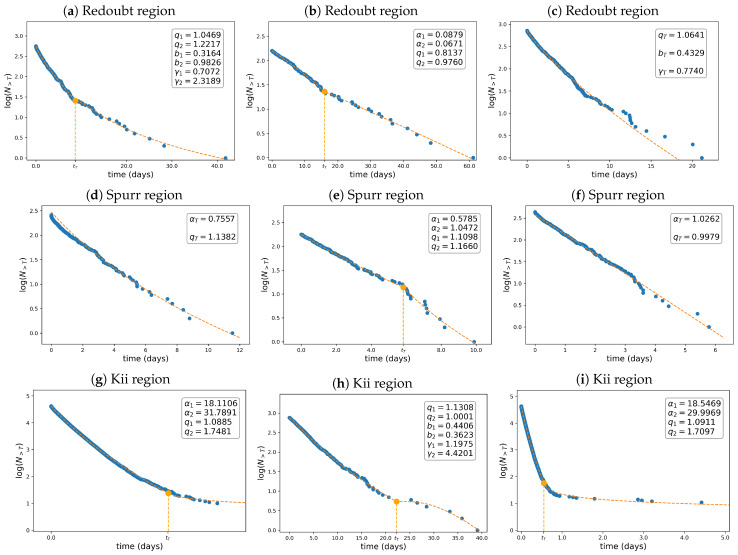
Interevent time distributions. The first, second, and third rows correspond to the waiting time distributions for the Redoubt, Spurr, and Kii regions, respectively. The distributions for shallow and deep events above $M_{0}=M_{c}$ are depicted in the first and second columns, respectively. In the third column, the interevent time distributions for all events are depicted. In all cases, the unimodal-bimodal Tsallis distribution has been fitted, except in (**a**), which shows the distribution for shallow events in the Redoubt area, modeled by a unimodal *q*-gamma distribution; (**c**), which shows the interoccurrence times for all events occurring in the Redoubt area, modeled by the *q*-gamma bimodal distribution; and (**h**) which shows the interevent time distribution of deep quakes in the Kii area. The standard deviations of the estimated parameters are summarized in Table 7.

Figure 8 depicts the interevent time distributions (waiting times) for all regions examined. The dashed orange curve represents the best fit, while the dots represent the data points. The standard deviations (measures of goodness) of the estimated parameters are summarized in Table 7. The first, second, and third rows refer to the Mount Redoubt, Mount Spurr, and Kii Peninsula regions, respectively, while the first, second, and third columns correspond to shallow, deep, and all (shallow + deep) events. We employed the *q*-gamma distribution to analyze the inter-occurrence times since it describes the behavior of inter-occurrence times well, as reported in [53]. This distribution is a generalization of the gamma distribution used by [59] for modeling interevent times. However, given that in the analyzed seismogenic areas, earthquakes are generated by several types of mechanisms represented by the different modes in the multimodal FMD, as previously discussed, we also expect to observe empirical multimodal distributions for the inter-occurrence time distributions. Thus, describing the interevent time distribution using the multimodal *q*-gamma distribution proposed in Section 2.3 is suitable. In this regard, we note that the *q*-values are generally greater than one, except for the Redoubt region when assessing the waiting times for shallow and deep earthquakes. In addition, the shape parameters have a similar order of magnitude for the volcanic areas, i.e., Mount Redoubt and Mount Spurr (∼$10^{-1}$). However, the Kii region’s shape parameters are considerably greater (∼10). Thus, the rate of events per unit of time in the Kii region is greater than that in volcanic areas. In other words, in the Kii Peninsula, waiting times are shorter than in the volcanic regions, possibly because the Kii region is larger and experiences more frequent earthquakes, as depicted in Figure 8i.

## 5. Conclusions

In this work, we proposed multimodal models in the context of non-extensive statistical mechanics to describe multi-source seismicity in terms of magnitude and spatial and temporal distribution. Our objective was to respond to the demand for new statistical models to describe multi-source seismicity, since classical models, which assume self-similarity, cannot fully explain this type of seismicity, which exhibits, at least, non-fractal behavior. Furthermore, our analysis of magnitude distributions revealed local behaviors that the Sotolongo–Costa Model, a generalization of the Gutenberg–Richter law that describes fractal phenomena, can interpret. We also observed evidence that earthquakes generated by a single source tend to present fractal behavior. Based on these considerations, we suggest that multi-source seismicity may exhibit multifractal behavior. However, this is a preliminary hypothesis that requires further investigation. Thus, provisionally, we assert that the observed behavior is at least non-fractal.

It is worth noting that the application of nonlinear and multifractal methods of analysis is of great benefit for understanding complex phenomena. For example, recent studies in mining have shown that acoustic and electromagnetic emissions in rock fractures exhibit multifractal properties and fracture propagation patterns similar to those in earthquakes. These analyses provide a refined understanding of fracture processes and fault propagation in rocks and provide indicators for predicting disasters in underground environments [60]. In addition, the use of variables such as seismic wave velocity and its gradient has been shown to be effective in detecting stress anomalies, which are essential for the assessment of risk areas [61]. Such approaches emphasize the relevance of nonlinear techniques and advanced statistical models that can provide a robust basis for monitoring seismic activity and complement traditional models, such as the Gutenberg–Richter Law, when dealing with the complexity inherent in seismological systems.

For the first time, a multimodal extension of the celebrated SCP model was applied to multi-source natural seismicity, surpassing the models found in the literature. In this context, the literature has explored the magnitude distribution in anthropogenic and natural multimodal seismicity, but there has been less focus on interevent distances and times. We found that behavior far from classical results was also observed for the interevent distance and time distributions, for which multimodal Tsallis and *q*-gamma distributions were successfully fitted. Real-world data analysis showed that our proposed model is workable. Further, our proposed model’s outstanding agreement with field data highlights a new way to study Earth’s seismicity, such as calculating earthquake recurrences. Additionally, multimodal models could be used to estimate seismic hazard, performing better than unimodal models, given that the fit of the former is tighter than that of the latter, especially for large quakes that can be underestimated or overestimated if unimodal models are employed.

In particular, the use of a multimodal model is relevant when determining the magnitude of completeness; this is because using a unimodal distribution generates inadequate values for the magnitude of completeness. Indeed, the proposed multimodal distribution, like the GR law, can help in seismic risk assessment and emergency response planning, among other applications, by more accurately estimating the expected frequency–magnitude of earthquakes, providing insights into the probability of specific magnitudes occurring. In addition, a more accurate earthquake magnitude frequency law contributes to seismic monitoring and early warning systems. Finally, this work can be complemented by future research considering earthquake source theory to improve the understanding of the various sources and their interactions. In addition, in volcanic areas, future studies should focus on the spatial delimitation between seismic sources near volcanoes (affected by magma) and the adjacent shallow crust (unaffected by magma). A detailed analysis based on focal mechanisms, for instance, would be essential to more accurately distinguish these sources, enhancing interpretation and seismic characterization at higher resolution.

## Figures and Tables

**Table 1:** Information on the analyzed catalogs: earthquake regions, the period covered by the datasets, and the number of events recorded for each region.

Region/Catalog	Period	Number of Events		
		Total	Shallow	Deep
Redoubt/USGS	2018–2022	1055	798	257
Spurr/USGS	2020	613	359	254
Kii/JMA	2013–2021	81,154	80,054	1100

**Table 2:** Estimated completeness magnitudes for the three seismic areas.

	Depth	Shallow	Deep
Region			
Redoubt		−0.6	1.45
Spurr		0.2	1.25
Kii		0.5	2.6

**Table 3:** Errors of the unimodal and multimodal fits for the F–M distribution. It can be observed from the root mean square error (RMSE), sum of squared errors (SSE), coefficient of determination ($R^{2}$), and mean absolute percentage error (MAPE) that, in all cases, the multimodal models fit more tightly than the unimodal ones.

Region	Events	Model	RMSE	SSE	$R^{2}$	MAPE (%)
Redoubt	All	Unimodal	0.0917	0.1223	0.9000	26.74
		Multimodal	0.0335	0.0167	0.9863	10.01
	Shallow	Unimodal	0.060	0.0507	0.9522	16.45
		Multimodal	0.0079	0.0010	0.9990	2.02
	Deep	Unimodal	0.0625	0.9265	0.9265	8.68
		Multimodal	0.0345	0.0133	0.9660	4.57
Spurr	All	Unimodal	0.0352	0.0167	0.9812	28.19
		Multimodal	0.0131	0.0021	0.9976	40.84
	Shallow	Unimodal	0.0218	0.0218	0.9944	42.94
		Multimodal	-	-	-	-
	Deep	Unimodal	0.0310	0.0310	0.0310	4.95
		Multimodal	0.0227	0.0056	0.9876	3.59
Kii	All	Unimodal	0.0452	0.0452	0.9511	4.34
		Multimodal	0.0203	0.0061	0.9907	1.83
	Shallow	Unimodal	0.0108	0.0018	0.9972	1.06
		Multimodal	-	-	-	-
	Deep	Unimodal	0.0360	0.0129	0.9762	3.31
		Multimodal	0.0134	0.0021	0.9960	1.95

**Table 4:** The standard deviations of the parameters shown in Figure 5.

Region	Events	$\sigma_{\alpha_{1}}$	$\sigma_{\alpha_{2}}$	$\sigma_{\alpha_{3}}$	$\sigma_{q_{1}}$	$\sigma_{q_{2}}$	$\sigma_{q_{3}}$	$\sigma_{M_{T}^{1}}$	$\sigma_{M_{T}^{2}}$
					$\cdot 10^{-2}$	$\cdot 10^{-2}$	$\cdot 10^{-2}$	$\cdot 10^{-2}$	$\cdot 10^{-2}$
Redoubt	All	$1.25\cdot 10^{-4}$	3.61·10	$3.81\cdot 10^{3}$	0.60	3.56	3.09	5.64	5.04
	Shallow	$6.74\cdot 10^{-5}$	$7.51\cdot 10^{-1}$	-	0.76	0.89	-	6.29	-
	Deep	$1.17\cdot 10^{-2}$	$3.63\cdot 10^{3}$	-	2.78	3.29	-	5.40	-
Spurr	All	3.04	3.97	$8.55\cdot 10^{3}$	4.98	1.11	2.26	4.92	63.1
	Shallow	$5.90\cdot 10^{-1}$	-	-	0.75	-	-	-	-
	Deep	$4.75\cdot 10^{-3}$	0	-	1.24	0.027	-	3.31	-
Kii	All	$1.75\cdot 10^{-3}$	$4.08\cdot 10^{-1}$	-	1.14	0.78	-	29.5	-
	Shallow	$4.59\cdot 10^{-1}$	-	-	0.35	-	-	-	-
	Deep	$1.17\cdot 10^{3}$	$7.48\cdot 10^{4}$	-	3.11	1.52	-	3.27	-

**Table 5:** Errors of fits shown in Figure 6 and Figure 8 for the interevent distances and times. It can be observed from the root mean square error (RMSE), sum of squared errors (SSE), coefficient of determination ($R^{2}$), and mean absolute percentage error (MAPE) the fits are very good.

Region	Events	Interevent	RMSE	SSE	$R^{2}$	MAPE (%)
Redoubt	All	Distances	-	-	-	-
		Times	0.0074	0.0024	0.9991	4.49
	Shallow	Distances	0.010	0.0045	0.9982	3.44
		Times	0.0088	0.0031	0.9987	5.48
	Deep	Distances	0.021	0.0083	0.9941	9.24
		Times	0.0155	0.0047	0.9966	7.52
Spurr	All	Distances	-	-	-	-
		Times	0.0101	0.0051	0.9977	6.78
	Shallow	Distances	0.0120	0.0043	0.9975	3.96
		Times	0.0845	0.1550	0.9138	19.36
	Deep	Distances	0.0182	0.0076	0.9948	6.10
		Times	0.0105	0.0025	0.9982	4.82
Kii	All	Distances	-	-	-	-
		Times	0.0193	0.1070	0.9938	6.64
	Shallow	Distances	0.0336	0.3859	0.9820	6.56
		Times	0.0203	0.1165	0.9932	6.96
	Deep	Distances	0.0136	0.0085	0.9972	7.44
		Times	0.0097	0.0048	0.9984	5.82

**Table 6:** The standard deviations of the parameters shown in Figure 6.

Region	Events	$\sigma_{\alpha_{1}}$	$\sigma_{\alpha_{2}}$	$\sigma_{q_{1}}$	$\sigma_{q_{2}}$	$\sigma_{r_{T}}$
		$\cdot 10^{-3}$	$\cdot 10^{-3}$	$\cdot 10^{-2}$	$\cdot 10^{-2}$	$\cdot 10^{-2}$
Redoubt	Shallow	1.00	7.67	0.43	3.01	7.64
	Deep	3.38	2.38	20.49	9.80	103.98
Spurr	Shallow	2.37	44.18	0.83	15.18	16.21
	Deep	1.14	7.28	2.28	9.91	103.83
Kii	Shallow	0.69	0.066	0.041	0.042	1.32
	Deep	0.10	4.02	0.48	51.28	46.55

**Table 7:** The standard deviations of the parameters shown in Figure 8. The main measures of goodness of fit are shown in Table 5. The parameters corresponding to the fits by q-gamma distributions are not shown.

Region	Events	$\sigma_{\alpha_{1}}$	$\sigma_{\alpha_{2}}$	$\sigma_{q_{1}}$	$\sigma_{q_{2}}$	$\sigma_{t_{T}}$
		$\cdot 10^{-3}$	$\cdot 10^{-3}$	$\cdot 10^{-2}$	$\cdot 10^{-2}$	
Redoubt	Shallow	2.58	3.29	2.24	2.08	0.28
Spurr	All	10.57	-	0.37	-	-
	Shallow	20.21	-	0.80	-	-
	Deep	13.79	93.33	1.10	3.08	0.066
Kii	All	8.62	0.11	0.015	0.41	17.48
	Shallow	8.85	1.91	0.016	0.25	2.52

## Data Availability

The data supporting the findings of this study are available from the corresponding author upon reasonable request.
